# Health care workers’ perspectives about disclosure to HIV-infected children; cross-sectional survey of health facilities in Gauteng and Mpumalanga provinces, South Africa

**DOI:** 10.7717/peerj.893

**Published:** 2015-04-14

**Authors:** Sphiwe Madiba, Mathildah Mokgatle

**Affiliations:** 1 Department of Environmental and Occupational Heath, School of Public Health, Sefako Makgatho Health Sciences University, Pretoria, South Africa; 2 Department of Biostatistics, School of Public Health, Sefako Makgatho Health Sciences University, Pretoria, South Africa

**Keywords:** Healthcare workers, South Africa, Disclosure, Disclosure guidelines, Perinatally infected children, Caregivers

## Abstract

The perspectives and practices of health care workers (HCWs) regarding disclosure to HIV-infected children have not been adequately investigated ten years after the roll-out of pediatrics antiretroviral therapy (ART). The aim of the study was to examine the opinions of HCWs about disclosure to HIV-infected children and determine their role in disclosure to children accessing ART in health centers in South Africa. This was a cross-sectional survey using a semi-structured questionnaire among HCWs in ART centers at three hospitals and 48 primary health facilities in two provinces in South Africa. Of the 206 HCWs, 140 (68.2%) were nurses, 44 (21.5%) were lay counsellors, and 4 (2%) were doctors. The majority (*n* = 183, 89.3%) felt that disclosure benefits children and they should be told about their HIV status. Over half (*n* = 93, 51.4%) recommended 11–18 years as the appropriate age to disclose. Half (*n* = 99, 48.5%) said that caregivers should take the lead to disclose, 87 (42.7%) said that disclosure is a shared responsibility of caregivers and HCWs, and 18 (8.8%) said HCWs should lead disclosure. HCWs perceived their role as that of preparing the caregiver for disclosure and the child to understand the disease. However, the lack of guidelines and training on disclosure counselling for children affects their ability to fully participate in disclosure to children. There is a need to adopt the World Health Organizations’ disclosure guidelines for children and adapt them to the local cultural and community contexts and train HCWs to guide, support, and assist caregivers in their disclosure to HIV-infected children.

## Introduction

Survival of perinatally infected children into adolescence and beyond made disclosure a major challenge for caregivers (Wiener et al., 2007) and healthcare professionals providing service to HIV-infected children (WHO, 2011). As a result, there are significant numbers of children and adolescents who are receiving treatment without being fully informed about their HIV status (Madiba, 2012; Spiegel, 2011).When full disclosure occurs, children are told the name of the illness (HIV and/or AIDS), disease specific information (how the virus is transmitted), and how they acquired the disease (Wiener et al., 2007). The American Academy of Pediatrics published disclosure guidelines in 1994 to promote disclosure to HIV-infected children (AIDS , 1999). However, in many developing countries, health care workers (HCWs) still lack the support of policies and guidelines on when and how children should be informed about their HIV status or their caregivers’ HIV status (WHO, 2011). While guidelines on disclosure of HIV status among adults have received considerable attention (Wiener et al., 2007), until the recent guidelines developed by the World Health Organization (WHO, 2011), there were no such guidelines for assisting HCWs to support caregivers to make decisions about disclosure to HIV-infected children in resource-limited settings (Moodley et al., 2006; Myer et al., 2006; Oberdorfer et al., 2006; Rujumba, Mbasaalaki-Mwaka & Ndeezi, 2010).

On the other hand, many caregivers are reluctant to inform their HIV-infected children about their status (Biadgilign et al., 2009; Butler et al., 2009; Funck-Brentano et al., 1997; Heeren et al., 2012; Mahloko & Madiba, 2012). One of the major barriers to disclosure is that caregivers, particularly from resource-limited settings, lack knowledge, skills, and guidance on how to approach disclosure to HIV-infected children (Madiba & Mokwena, 2012; Mahloko & Madiba, 2012; Oberdorfer et al., 2006). It is argued that for disclosure to occur, caregivers have to first trust in their own readiness and ability to disclose (Dematteo et al., 2002; Madiba & Mokwena, 2012). Thus, caregivers need considerable support during the process of disclosure to children, often expressed by some of the caregivers from sub-Saharan Africa (Brown et al., 2011; Heeren et al., 2012; Madiba & Mokwena, 2012; Moodley et al., 2006; Oberdorfer et al., 2006; Vaz et al., 2010). The World Health Organization recommends that health services need to provide strategies that will allow HCWs to support caregivers to disclose to their HIV infected children by the age of 12 years (WHO, 2011). It has been reiterated by HCWs that, where caregivers lacked knowledge and skills to disclose, health services should adopt a disclosure program that would allow them to support caregivers to disclose to HIV-infected children (Kallem et al., 2011). Furthermore, it has been shown that caregivers who discuss disclosure with HCWs are more likely to disclose HIV status to their children (Merzel, VanDevanter & Irvine, 2008; Mumburi et al., 2014).

However, studies conducted on disclosure to HIV-infected children have focused on the experiences of caregivers, but the perspectives and practices of HCWs regarding disclosure have not been adequately investigated (Fair & Walker, 2011; Rujumba, Mbasaalaki-Mwaka & Ndeezi, 2010). Recent data from a situational analysis for pediatric HIV/AIDS care in Ethiopia (Rujumba, Mbasaalaki-Mwaka & Ndeezi, 2010) show that HCWs are still constrained by inadequate knowledge about pediatric HIV care as well as lack of knowledge of pediatric counselling. Fair & Walker (2011) argue that to fully understand disclosure to HIV-infected children, it is essential to understand the perspectives of all HCWs involved in the disclosure process. The purpose of the study was to assess how disclosure to HIV-infected children is being implemented in public health facilities. We examined the opinions of health care workers about disclosure to HIV-infected children and determined their role in disclosure to children accessing ART in primary health facilities in South Africa. There are limited studies on the perspectives and practices of HCWs on disclosure to HIV-infected children almost ten years after the roll-out of pediatric ART in South Africa and other sub-Saharan countries. Interventions to facilitate disclosure to HIV-infected children should incorporate the experiences and views of caregivers, HCWs, and infected children.

## Methods and Materials

### Study design

This cross sectional survey was part of a larger mixed-method study conducted to assess how disclosure to HIV-infected children is being implemented. The study was conducted among HCWs who provide HIV treatment and care services for adults and children in the ART centers of selected hospitals and primary health facilities. This paper reports on the quantitative data collected using semi-structured interviews but exclude qualitative data collected through focus group discussions to explore how disclosure is being implemented. HCWs who participated in focus group discussions were purposely selected because they had some experience in disclosing to children or having assisted caregivers to disclose, while all other HCWs participated in the survey.

Since 2011, ART has been accessible in primary health care facilities in South Africa through the Nurse Initiated and Managed Antiretroviral Treatment initiative of the Department of Health (Cameron et al., 2012). The HIV services provided through this initiative included adult and pediatric counselling and testing and initiation of ART. The study covered two districts in two provinces in South Africa: Tshwane district in Gauteng province and Nkangala District in Mpumalanga province. Data were collected in ART centers based in an academic hospital and two community hospitals, as well as 47 primary health care facilities. Of these, 17 were primary health centers and 20 were eight-hour clinics. The health facilities in Tshwane district were located in urban and peri-urban communities, while those in Nkangala district were located in three sub-districts, one urban and two rural. The study participants included medical doctors, nurses (professional, enrolled and assistant nurses), social workers, lay counsellors, pharmacists, and pharmacy assistants. In order to gather the opinions of all HCWs who come in contact with HIV-infected children and their caregivers, the sample consisted of all HCWs in each primary health facility and ART center at the time of data collection. Primary health facilities are generally understaffed in South Africa, and most of the eight-hour clinics, particularly in the rural districts of Mpumalanga province, have on average two professional nurses per shift. There were also no resident doctors, social workers, or psychologists in the Mpumalanga health facilities. There are, however, doctors and a psychologist available for consultation on cases that may be HIV-related or any other condition that needs the attention of other members of the multidisciplinary team. To limit selection bias, we collected data from HCWs across all shifts, but excluded those who were on leave. Data collection at the hospitals included only HCWs working in ART clinics.

### Data collection

We collected data between January and September, 2013. Fieldwork was led by the second author, who oversaw the training of seven field workers and the implementation of study activities (data collection, cleaning, and coding). The tool consisted of structured close-ended questions and a set of open-ended questions. Close-ended questions captured information about the participant’s demographic characteristics, whether the child should be told about their HIV status, the appropriate age of disclosure, who should disclose, whether they received training in disclosure counselling, and about the availability of disclosure guidelines in their facilities. The open-ended questions captured information on their views on why it was important to disclose to infected children, why caregivers delay disclosure, their role in disclosure, and the support they need to disclose to HIV-infected children. We used semi-structured questionnaires because we wanted to capture the specific variables from the perspectives of the HCWs as there was no prior research conducted on the topic to inform development of a quantitative tool. The questionnaires were in English and were distributed to the health facilities at the beginning of a shift and collected at the end of a shift. In analysis, the open ended responses were quantified and are presented in tables. All analysis were computed using Stata version 13.

### Ethics

Ethical clearance was obtained from the Medunsa Research Ethics Committee (MREC/H/168/2012: IR) of the University of Limpopo. In addition, permission to conduct the study was obtained from relevant authorities from the two provinces as well as the management of the hospitals and primary health facilities. Informed consent was obtained from healthcare workers.

## Results

### Description of study participants

Of the 206 HCWs who participated in the survey, the majority (*n* = 185, 90.2%) were female and the mean age was 41.3 years (range 20–64 years). Professional nurses constituted half (*n* = 103, 50.2%) of the HCWs, 44 (21.5%) were lay counsellors, 37 (18%) were enrolled and assistant nurses, 16 (7.8%) were pharmacists and pharmacist assistants, and 4 (2%) were doctors. The mean time of employment was 6.5 years, and three quarters (*n* = 117, 71.3%) reported that they treat adults and children infected with HIV daily (Table 1).

**Table 1:** Characteristics of healthcare workers and opinions about disclosure to HIV-infected children in primary health facilities and ART.

	Frequency	Percentage
**Gender**		
Female	185	90.2
Male	20	9.8
**Age category**		
20–30 years	22	10.8
31–40 years	64	31.4
41–50 years	83	40.7
51–60 years	33	16.2
61–70 years	2	1.0
Mean age 41.3 years		
**Professional qualifications**		
Professional Nurse	103	50.2
Lay Counsellor	44	21.5
Assistant Nurse	23	11.2
Enrolled Nurse	14	6.8
Pharmacist	11	5.4
Pharmacist Assistant	5	2.4
Medical Doctor	4	2.0
Social worker	1	0.5
**Disclosure guidelines available**		
No	123	76.9
Yes	37	23.1
**Received training on disclosure**		
No	133	82.1
Yes	29	17.9
**Children should be told about their HIV status**		
No	22	10.7
Yes	183	89.3
**Age of disclosure**		
5–7 years	26	14.4
8–10 years	62	34.2
11–14 years	64	35.4
15–18 years	29	16.0
Mean age 10.9 years		
**Right time for disclosure**		
When the child enters teenage	2	1.0
At puberty	51	26.4
When the child can understand	79	40.9
When the child is mature enough	32	16.6
At school age	29	15.0
**Appropriate person to disclose**		
Parent/caregiver	99	48.5
Parent/caregiver and health provider	87	42.7
Health care provider	18	8.8

### Healthcare workers’ perceptions about telling children that they have HIV

The majority (*n* = 183, 89.3%) said HIV-infected children should be told about their HIV status. HCWs were also asked an open-ended question about why it was important to tell children about their HIV status; their responses are presented in Table 2. The most common cited reasons for disclosure was for children to adhere to ART (*n* = 51, 21.7%), know their status (*n* = 55, 23.5%), take responsibility for their treatment and care (*n* = 36, 15.3%), understand the disease (*n* = 33, 14%), understand the reasons for taking medication (*n* = 30, 12.8%), protect others from HIV infection (*n* = 18, 7.7%), and live a positive and healthy life (*n* = 12, 5.1%).

**Table 2:** Healthcare workers’ perceptions about telling children that they have HIV.

	Frequency	Percentage
So that children should adhere to medication	51	21.7
To allow children to know their HIV status	49	20.9
For children to take responsibility for their own treatment and care	36	15.3
For children to understand the disease (HIV)	33	14.0
To know the reasons why they are taking medication	30	12.8
To protect others from being infected with HIV	18	7.7
To live a healthy life	12	5.1
Children have a right to know their disease	6	2.6

### The right age and time for telling children that they have HIV

The healthcare workers were asked about the right age to disclose to HIV-infected children and over a third (*n* = 64, 35.4%) said the child should be told between 11 and 14 years, 62 (34.2%) said between 8 and 10 years, 29 (16%) said between 15 and 18 years, and 26 (14.4%) said between 5 and 7 years. The results showed that over half (*n* = 93, 51.4%) cited an older age of above 10 years as the right age to tell children about their HIV status. With regards to the right time to tell the child about their disease, 79 (40.9%) said the child should be told when he/she could understand the disease and its implications, 51 (26.4%) said puberty was the right time to tell the child, 32 (16.6%) said the child should be mature enough at disclosure, and 29 (15%) said the child should be told at school going age (Table 1).

### The right persons to tell children that they have HIV

Almost half (*n* = 99, 48.5%) said that the caregivers are the relevant and appropriate people to disclose to children; 87 (42.7%) said that disclosure to HIV-infected children is a shared responsibility of the caregivers and the HCWs. However, the role of the HCWs in this regard was to support the caregiver’s disclosure, but not to lead or initiate disclosure. A few (*n* = 18, 8.8%) said that HCWs should lead and initiate disclosure; nurses, doctors, psychologists, social workers, and lay counsellors were the categories of HCWs mentioned (Table 1). With regards to why caregivers should take the lead in disclosure, 96 (47%) said due to the relationship between child and caregiver, the caregiver is better placed to monitor the child’s reaction to the disclosure, 26 (12.7%) said that the child trusts the caregiver, 8 (3.9%) said that the caregiver knows the right age to disclose, 7 (3.4%) said that the caregiver will support the child to adhere to the prescribed treatment plan, 6 (2.9%) said that the caregiver will support the child to cope with disclosure, and 8 (3.9%) said the child will be comforted if the caregiver discloses (Table 3).

**Table 3:** The perceptions of healthcare workers on why the caregiver or healthcare workers should take the lead in disclosure.

	Frequency	Percentage
**Caregivers should lead disclosure**		
The caregiver/parent is close to the child and is always there to monitor the child’s reaction to disclosure	96	47.1
The child trusts the caregiver/parent and will will accept the reality of their condition if the caregiver discloses	26	12.7
The caregiver/parent knows the right age to tell and knows what and how to tell the child about the disease	8	3.9
The caregiver/parent is always there to help the child to understand the importance of taking medication and support the child’s adherence to prescribed treatment plan	7	3.4
The caregiver/parent is always there to give the child emotional support to cope with disclosure	6	2.9
The caregiver/parent is always there and the child will be comfortable if the caregiver/parent is the one who discloses	8	3.9
**Healthcare workers should lead disclosure**		
HCWs are qualified and skilled and should take the lead in disclosing	35	17.2
HCW gives on-going support to the child and parents/caregiver	13	6.4
HCW gives on-going counselling to the child and parents/caregiver	5	2.5

### The reasons why caregivers delay telling children that they have HIV

The most cited reasons given by HCWs as to why caregivers delay disclosure were fear of hurting the child (*n* = 33, 18.6%), fear that the child would be angry, hate, blame, judge, and reject them (*n* = 31, 17.5%), that the child is too young and cannot understand the HIV diagnosis (*n* = 28, 15.8%), fear of the stigma related to HIV/AIDS (*n* = 25, 14.1%), that parents blame themselves and feel guilty about infecting the child (*n* = 20, 11.3%), caregivers are not ready to disclose and therefore it is not the right time to disclose (*n* = 20, 11.6%), and (*n* = 19, 10.7%) caregivers’ lack of experience in disclosure (Table 4).

**Table 4:** Healthcare workers’ perceptions about the reasons caregivers delay disclosure to infected children.

	Freq.	Percent
Fear of hurting the child	33	18.6
Afraid that the child will be angry and/or hate, blame, judge, and reject the parents	31	17.5
Child is too young and can’t understand HIV	28	15.8
Afraid of the stigma related to HIV	25	14.1
Caregivers are not ready to disclose and it is not the right time to disclose	20	11.3
Parents blame themselves and feel guilty about infecting the child	20	11.3
Caregivers don’t know how to disclose	19	10.7
Caregivers are afraid to disclose	15	8.5
Caregivers lack in-depth HIV related information	10	5.6
Afraid that the child will react negatively to disclosure and will be confused	8	4.5
Afraid to answer questions about HIV	7	4.0
Caregivers lack support to disclose	5	2.8
Afraid the child will tell others about their HIV diagnosis	3	1.7

### The role of health workers in disclosing to HIV-infected children

The healthcare workers were asked to identify their roles in disclosing to HIV-infected children who access ART in their health facilities. The most common roles mentioned included supporting caregivers to disclose and helping children to accept their status (*n* = 65, 38.2%), providing continuous health education to children (*n* = 31, 18.2%), providing ongoing counselling to caregivers and children (*n* = 31, 18.2%), educating the child about the disease (*n* = 30, 17.6%), ensuring that the child adheres to treatment (*n* = 18, 10.6%), and providing information to caregivers and children (*n* = 17, 10%). Only 4 (2.4%) said that their role was to disclose to HIV-infected children (Table 5).

**Table 5:** The roles of healthcare workers in the process of disclosing to HIV-infected children.

	Freq.	Percent
Support the caregiver through the disclosure process and the child after disclosure to live a healthy life	65	38.2
Provide health education to children to take care of themselves	31	18.2
Provide ongoing counselling to caregivers to manage disclosure and to children so that they accept their status	31	18.2
To ensure that after disclosure the child understands HIV and treatment	30	17.6
Provide ART and ensure that the child adheres to treatment	18	10.6
Provide information about the importance of disclosure to assist caregivers to disclose	17	10.0
Provide HIV-related information after disclosure so that the child understands the disease and the importance of adherence to medication	17	10.0
Facilitate and initiate disclosure to the child when the time is right to disclose	10	5.9
Monitor the reaction of the child after disclosure	5	2.9
Answer questions that the child and caregiver ask about HIV during disclosure	5	2.9
Prepare the child for the process of disclosure	4	2.4
Encourage caregivers to disclose	4	2.4
Assist caregivers to disclose	4	2.4

### The support needed by HCWs to facilitate disclosure to HIV-infected children

Over three quarters (*n* = 123, 76.9%) reported that there were no guidelines on disclosure counselling for children in their health facilities, and 133 (82.1%) did not receive any formal training on disclosure counselling for children. HCWs also responded to a question that asked them how they would like to be supported to participate in the disclosure process for HIV-infected children. The majority (*n* = 84, 40.8%) reported that they need to be trained on disclosure counselling for children, 49 (23.8%) need to attend workshops on pediatric HIV management, 35 (17%) need formal guidelines on disclosure counselling for children, and 13 (6.3%) need ongoing counselling and debriefing to deal with HIV-infected children (Table 6).

**Table 6:** Support needed by healthcare workers to facilitate disclosure to children in primary health facilities.

	Freq.	Percent
In-service education and training on disclosure counselling to support HIV-infected children to understand the disease	84	40.8
Workshops and training on HIV management to get skills and increase their confidence in assisting caregivers to disclose	49	23.8
Guidelines on disclosure counselling for children	35	17.0
Counselling to be able to deal with HIV-infected children	13	6.3

## Discussion

This study examined the perspectives of healthcare workers about disclosure to HIV-infected children ten years after the initiation of ART in South Africa. The majority felt that HIV-infected children should be told about their HIV status but believed that disclosure depends on the age and maturity of the child. Research shows that caregivers of HIV-infected children have similar views about disclosure to HIV-infected children (Kiwanuka, Mulogo & Haberer, 2014; Motshome & Madiba, 2014; Vaz et al., 2010). Healthcare workers also stated that children benefit from disclosure because it gives them an understanding of their condition. Telling children that they are HIV-positive is also crucial for their understanding of the importance of treatment adherence and acceptance of their HIV status. When children and adolescents know their status, they can learn to protect others and themselves from HIV infections. Healthcare workers in Kenya cited similar benefits of disclosure to HIV-infected children (Beima-Sofie et al., 2014).

Over half of the healthcare workers suggested an older age of above 10 years as the right age to tell children about their status. They believed that children are mature enough around this age and they may be, or may become sexually active and risk reinfection with a different strain of the virus, transmitting the virus to a sexual partner or becoming resistant to treatment. Those who suggested a younger age for disclosure (below 10 years), believed that this is the time when children start asking questions about their disease, are curious about taking medication continuously, and could have a general understanding about HIV infection and transmission in order to protect others from HIV infection. In a previous study conducted in South Africa, HCWs also mentioned that children should be informed about their HIV status when they start formal schooling (Myer et al., 2006).

The data suggest that the recommended age of disclosure to children is subjective and is likely to be influenced by community and social contexts of disclosure. In many communities in poor resourced countries, HIV-related stigma and discrimination, secrecy, and fear of death and dying influence disclosure across all population groups (Biadgilign et al., 2009; Kiwanuka, Mulogo & Haberer, 2014; Madiba & Mokwena, 2012). The current findings showed a relatively older recommended age (above 10 years, range 11–18 years) of disclosure as compared to an age of 6 years that was suggested by HCWs in a much earlier study conducted in South Africa (Myer et al., 2006). However, healthcare workers in a recent study in South Africa recommended 12 years as the age of disclosure to HIV-infected children (Heeren et al., 2012). The fact that caregivers continue to delay disclosure to HIV-infected children despite children being on ART influenced the perceptions of HCWs about disclosure to infected children. On the other hand, HCWs are members of communities where HIV-related stigma is still a barrier to disclose the HIV status of infected adults and children.

Healthcare workers in the current study and others, viewed telling the child about their HIV status as the responsibility of the caregiver (Kallem et al., 2011; Kidia et al., 2014; Mumburi et al., 2014; Myer et al., 2006). A disclosure intervention developed by Salter-Goldie and colleagues also recommends that caregivers take the lead in disclosure, and should plan where, when, with whom and what will be said during disclosure (Salter-Goldie et al., 2007). In the current study, HCWs argue that because disclosure depends on the child’s age and ability to understand, the caregiver knows when the child is ready for disclosure. They perceived their role in the disclosure process as that of providing additional information and explanations about HIV as well as ongoing support and counselling to the caregiver and the child. Our data supports current findings from a study conducted in Kenya (Beima-Sofie et al., 2014).

Healthcare workers who were of the opinion that telling children about their HIV status is a shared responsibility, believed that caregivers need their assistance to tell children about their HIV status because disclosure is a difficult task for the caregiver. The majority of HCWs, particularly nurses, see their role in this regard as that of preparing the caregiver for disclosure and to help the child to understand the disease and adhere to ART. These roles are similar to what have been reported in a disclosure intervention that involved a health care team’s approach to disclose to infected children (Salter-Goldie et al., 2007).

Consistent with previous studies, only a few HCWs said that the actual procedure of telling children about their HIV status is their responsibility (Heeren et al., 2012; Myer et al., 2006), and nurses, doctors, psychologists, social workers, and lay counsellors were the categories of HCWs that were mentioned. They maintain that HCWs can explain HIV better than the caregivers, that they will be able to prepare the children psychologically before disclosure, and that they were in a better position to deal with negative reactions to disclosure because they are qualified professionals with adequate knowledge and counselling skills.

The involvement of HCWs in disclosure to HIV-infected children has been steadily increasing according to data from sub-Saharan countries (Heeren et al., 2012; Madiba, 2012; Mahloko & Madiba, 2012; Rujumba, Mbasaalaki-Mwaka & Ndeezi, 2010; Vaz et al., 2010), despite the lack of training on disclosure counselling of children (Beima-Sofie et al., 2014). Consistent with previous studies, one of the major concerns of HCWs was the lack of formal guidelines on child counselling to guide them on when and how to prepare and support caregivers to disclose to children (Myer et al., 2006; Rujumba, Mbasaalaki-Mwaka & Ndeezi, 2010). We found that the recent published World Health Organization disclosure guidelines for children have not yet been adopted and utilized by HCWs in all the health facilities. Moreover, HCWs in the current study and in many sub-Saharan countries are hardly ever trained in pediatric HIV and in disclosure counselling of children, and lacked skills to assist caregivers to disclose (Rujumba, Mbasaalaki-Mwaka & Ndeezi, 2010). Attending workshops and receiving training will give HCWs skills and increase their confidence in assisting caregivers to disclose but also support HIV-infected children to understand the disease (Beima-Sofie et al., 2014; Kallem et al., 2011; Rujumba, Mbasaalaki-Mwaka & Ndeezi, 2010; Wiener et al., 2007).

While the lack of training and formal guidelines on child counselling and pediatric HIV are major constraints in disclosing to children, caregivers’ fears and concerns also play a crucial role in delaying disclosure. Consistent with findings from other studies, HCWs were of the views that caregivers delay disclosure because they believe that when children learn about their HIV status they will be hurt. One other common reason cited by HCWS was that caregivers delay disclosure because they believe that the child is too young to understand HIV/AIDS (Rujumba, Mbasaalaki-Mwaka & Ndeezi, 2010; Kidia et al., 2014). Madiba & Mokwena (2012) found that when caregivers say that the child is too young, they also mean that the child is unable to understand the negative consequences of an HIV diagnosis. Age was often not used in determining the child’s ability to understand HIV/AIDS because it was subjective. This point of view was apparent in the current study where HCWs recommended an older age of above 10 years as the right age to tell children about their HIV status. The data suggest that age is not necessarily the determining factor for disclosure and should not be used to guide parents and caregivers in the decision to disclose HIV status to children.

Other reasons cited by HCWs as to why caregivers delay disclosure to children were similar to previous findings from studies conducted with caregivers. These included fear of stigmatization and discrimination, lack of disclosure skills, self-blame and guilt for infecting the child with HIV, and fear of being rejected by the child (Beima-Sofie et al., 2014; Biadgilign et al., 2009; Kallem et al., 2011; Madiba & Mokwena, 2012; Mahloko & Madiba, 2012; Motshome & Madiba, 2014; Vaz et al., 2011; Vreeman et al., 2014). HCWs also stated that caregivers often delay disclosure because the caregiver is not ready to disclose. Madiba & Mokwena (2012) argue that caregiver readiness is the determining factor in disclosure. Thus, when a caregiver is not ready to disclose, they will simply avoid informing the child of their HIV status. There is a need to develop appropriate disclosure interventions to address caregiver’s deep seated fears of disclosing to their HIV-infected children (Vreeman et al., 2013).

### Limitations

Because of the limited involvement of HCWs in disclosure, the data presented here represent their opinions rather than their practice of disclosure. The majority made recommendations on what HCWs who are involved in disclosure should do. Nevertheless, these study findings have revealed the current beliefs and views about disclosure to HIV-infected children among HCWs in primary health facilities. In addition, these findings form a baseline understanding about disclosure from the perspectives of HCWs, which will inform the development of interventions to facilitate disclosure to children.

## Conclusion

Healthcare workers believed that children should be told about their status and argue that the disclosure process should facilitate a child’s understanding and acceptance of living with the disease. However, the recommended age of disclosure was set much higher than previously documented in South Africa. The healthcare workers’ attitudes towards the right time to disclose might be influenced by the cultural and social contexts of disclosure within their communities.

To ensure that the outcome of the disclosure process is positive, they believed that the caregiver should take the lead in disclosure while being supported by the HCW. However, they pointed out that lack of guidelines and training on disclosure counselling of children affects their ability to participate fully in disclosure which often results in delayed disclosure to children. It is expected that when they are trained and participate fully in disclosure, children can be informed of their HIV status in an appropriate, sensitive manner.

To facilitate disclosure, adoption of the World Health Organization disclosure guidelines for children and their adaptation to the local cultural and community contexts is crucial. These guidelines should form the basis for training of HCWs to equip them with appropriate skills to support caregivers in disclosing HIV status to children and to ensure that they are counselled suitably to accept their condition.

## Supplemental Information

10.7717/peerj.893/supp-1 Supplemental Information 1 Data set
